# The association of asthma and its subgroups with osteoporosis: a cross-sectional study using KoGES HEXA data

**DOI:** 10.1186/s13223-020-00482-6

**Published:** 2020-09-25

**Authors:** Jee Hye Wee, Chanyang Min, Min Woo Park, Soo Hwan Byun, Hyo-Jeong Lee, Bumjung Park, Hyo Geun Choi

**Affiliations:** 1 Department of Otorhinolaryngology-Head and Neck Surgery, Hallym University Sacred Heart Hospital, 22, Gwanpyeong-ro 170 beon-gil, Dongan-gu, Anyang, 14068 Republic of Korea; 2 Hallym Data Science Laboratory, Hallym University College of Medicine, Anyang, Korea; 3 Graduate School of Public Health, Seoul National University, Seoul, South Korea; 4 Department of Otorhinolaryngology-Head and Neck Surgery, Kangdong Sacred Heart Hospital, Seoul, South Korea; 5 Department of Oral and Maxillofacial Surgery, Dentistry, Hallym University College of Medicine, Anyang, South Korea

**Keywords:** Asthma, Osteoporosis, Chronic disease, Epidemiology

## Abstract

**Background:**

A few studies have reported the association between asthma and osteoporosis. We aimed to analyze the association of asthma and its subgroups with osteoporosis in the Korean adult population.

**Methods:**

We used the health examinee (HEXA) data from the Korean Genome and Epidemiology Study (KoGES) obtained between 2004 and 2016. We included 162,579 participants (n = 3,160 with asthma; n = 159,419 controls) who reported their previous histories of asthma and osteoporosis. The participants were categorized into 3 groups based on asthma management: participants who did not need further treatment due to controlled symptoms (well controlled); participants with ongoing treatment (being treated); participants who were not treated even though they had symptoms (not being treated). Multiple logistic regression analyses were used to calculate the adjusted odds ratios (aORs) with 95% confidence intervals (CIs) for osteoporosis. Subgroup analyses for age and sex were conducted.

**Results:**

The prevalence of osteoporosis was higher in patients with asthma (13.6%) than in controls (6.8%). In the full-adjusted model, the aORs for osteoporosis were 1.74 (95% CI 1.55–1.94, P < 0.001) in patients with asthma compared to controls. There were consistent findings across the age and sex subgroups. The aORs for osteoporosis were 1.43 (95% CI 1.10–1.86, P = 0.008) in the well-controlled asthma group; 1.55 (95% CI 1.28–1.89, P < 0.001) in the being treated asthma group; and 1.96 (95% CI 1.66–2.31, P < 0.001) in the not being treated asthma group compared to the control group.

**Conclusion:**

Asthma was associated with osteoporosis in the Korean adult population. Patients with asthma not being treated showed the highest ORs for osteoporosis.

## Background

Osteoporosis is characterized by low bone mass and bone fragility, which increases the risk of bone fracture [1]. The prevalence of osteoporosis among adults aged ≥ 50 years in the USA was estimated at 16.0% and 29.9% in men and women, respectively [2]. The Korean National Health and Nutrition Examination Survey conducted between 2008 and 2011 reported that the prevalence of osteoporosis in the Korean population aged ≥ 50 years was 7.3% and 38.0% in males and females, respectively [3].

Asthma is the most prevalent chronic inflammatory airway disease, which involves high morbidity and mortality rates in severe cases. It is important to recognize the comorbidities since these can complicate the clinical treatment of patients with asthma in various ways. Asthma has been reported to be associated with several other chronic diseases, including diabetes, metabolic syndrome, cardiovascular disease, rheumatoid arthritis, and psychiatric disease [4–8]. In Scotland, a population-based cross-sectional analysis reported that 62.6% and 46.23% of adults with and without asthma, respectively, had ≥ 1 comorbidity; moreover, 16.3% and 8.7% of adults with and without asthma, respectively, had > 4 comorbidities [9]. The etiology of asthma co-morbidities could be associated not only with asthma, but with other morbidities and common mechanisms, as well as environmental and genetic risk factors [4, 10].

Recent studies have demonstrated the role of systemic inflammatory response seen in chronic airway disease in osteoporosis development [11–13]. Inflammatory cytokines, including tumor necrosis factor (TNF)-α and interleukin (IL)-6, act as osteoclast stimulants, which promotes bone resorption. However, a few studies have reported the association of asthma itself with osteoporosis. A cross-sectional healthcare center-based study on Korean adults reported that the proportion of patients with osteopenia or osteoporosis was much higher in the airway hyper-responsiveness (AHR)-positive group than in the AHR-negative group (odds ratio [OR] = 1.715, 95% confidence interval [CI] 1.252–2.349), as well as in the ever-asthma group than in the never-asthma group (OR = 1.526, 95% CI 1.120–2.079), except in patients with a history of systemic corticosteroid use [14]. A cross-sectional hospital-based study on USA adults showed that the emergency department visit of patients with versus without asthma were associated with higher odds of osteoporosis (1.85, 95% CI 1.82–1.88) [15]. However, the studies involved a small sample size or were hospital-based analyses.

We aimed to analyze the association between asthma and osteoporosis in the Korean adult population. Further, we aimed to determine the different effects of asthma on osteoporosis based on the condition of asthma management. The management of patients with asthma was classified as well controlled, being treated, and not being treated.

## Methods

### Study population and data collection

The ethics committee of Hallym University (2019-02-020) approved the use of these data. The requirement for written informed consent was waived by the Institutional Review Board. This cross-sectional study used the data of Health Examinee (HEXA) population-based cohort among the Korean Genome and Epidemiology Study (KoGES), which is a consortium project consisting of six prospective cohort studies (i.e., the population-based cohorts: the community-based KoGES_Ansan and Ansung study, the urban community-based KoGES_health examinee (HEXA) study, and the rural community-based KoGES_cardiovascular disease association study (CAVAS); the gene-environment model studies: the KoGES_twin and family study, the KoGES_immigrant study and KoGES_emigrant study). The KoGES HEXA study included participants aged ≥ 40 years old who visited the institutions, which are mainly general hospitals in the metropolitan areas and major Korean cities. The data comprised of baseline data obtained in 39 sites from 2004 to 2013 and follow-up data obtained from 2012 to 2016. Details on data collection have been previously described [16].

### Participant selection

**Fig. 1:**
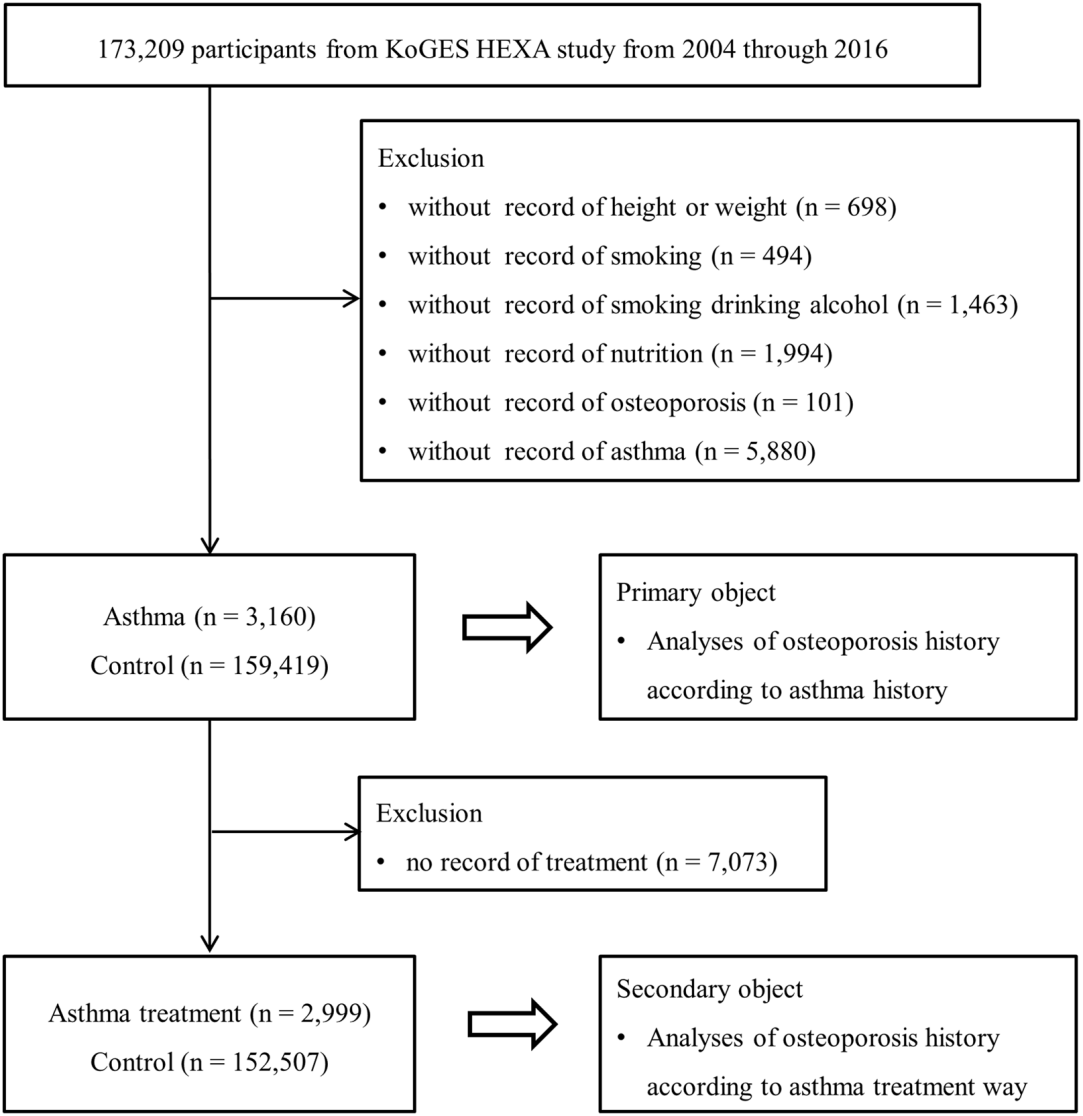
A schematic illustration of the participant selection process that was used in the present study. A total of 162,579 participants were enrolled

Among the 173,209 participants, we excluded participants with missing records regarding height or weight (n = 698), smoking history (n = 494), drinking alcohol habit (n = 1463), nutrition records (n = 1994), osteoporosis (n = 101), and asthma history (n = 5880). The number of missing records of asthma history are many because asthma was not surveyed in 2004. Finally, we included 3160 patients with asthma and 159,419 controls (with no history of asthma) (Fig. 1). Subsequently, we performed a between-group analysis of the history of osteoporosis (primary objective). Further, we analyzed the history of osteoporosis according to the condition of asthma management (secondary objective). We excluded 7073 participants from both groups for lacking treatment records obtained in 2008.

### Survey

Trained interviewers obtained information regarding the participants’ previous histories of asthma and osteoporosis. If the participants answered ‘yes’ to the question, they were then asked about the current status of management. The participants were categorized into the following three groups based on the status of asthma management: participants who were told by a medical doctor that medical treatment was not required due to resolution of symptoms (well controlled); participants undergoing treatment (being treated); and participants with untreated symptoms (not being treated). The body mass index (BMI) was calculated as kg/m^2^ using the measured height and weight values. Smoking history was categorized as non-smoker (< 100 cigarettes on entire life), past smoker (quit for > 1 year), and current smoker. Drinking alcohol habits were categorized as non-drinker, past drinker, and current drinker. Nutritional intake (Total calories [kcal/day], Protein [g/day], Fat [g/day], Carbohydrate [g/day], Calcium [mg/d], Phosphorus [mg/d], and Potassium [mg/d]) was surveyed using a food-frequency questionnaire, which was previously validated [17]. Income groups were categorized according to their household incomes as follows: non-respondent, low income (< ~$2000 per month), middle income (~ $2000–$3999 per month), and high income (~ ≥ $4000 per month).

### Statistical analyses

A Chi square test was used to compare the sex proportion, income group, smoking status, and drinking alcohol history. An independent *T* test was used to compare age, BMI, and nutritional intake. A logistic regression model was used to analyze the OR of asthma for osteoporosis as the primary objective. Moreover, we employed a crude and adjusted model (age, sex, income group, BMI, smoking, alcohol consumption, and nutritional intake [total calories, protein, fat, carbohydrate, calcium, phosphorus, and potassium]). In the age-based subgroup analyses, the median age was determined by using the dividing point (< 53 years old and ≥ 53 years old). Regarding the secondary objective, a logistic regression model was used to calculate the ORs of asthma treatment status (well controlled, being treated, and not being treated compared to the controls) for osteoporosis.

Two-tailed analyses were conducted with statistical significance set at P < 0.05. Statistical analyses were performed using SPSS v. 24.0 (IBM, Armonk, NY, USA).

## Results

Table 1 presents the general characteristics of the participants. Patients with asthma were more likely to be older, female, have higher BMI, lower income, non-smokers, and non-alcohol drinkers (all P < 0.001). Among the nutritional factors, there was a significant between-group difference in the amount of total calories (P = 0.002), protein (P < 0.001), fat (P < 0.001), and phosphorus (P = 0.003). The prevalence of osteoporosis was higher in participants with asthma (13.6%) than in controls (6.8%) (P < 0.001).

**Table 1:** General characteristics of participants

Characteristics	Total participants		P-value
	Asthma	Control	
Age (mean, SD, y)	55.9 (8.7)	53.2 (8.4)	< 0.001^a^
Sex (n, %)	24.4 (3.3)	23.9 (2.9)	< 0.001^a^
Men	952 (30.1)	54,772 (34.4)	
Women	2208 (69.9)	104,647 (65.6)	
BMI (mean, SD, kg/m^2^)	24.4 (3.3)	23.9 (2.9)	< 0.001^a^
Income (n, %)			< 0.001^a^
Missing, no response	471 (14.9)	20,322 (12.7)	
Lowest	1125 (35.6)	44,950 (28.2)	
Middle	996 (31.5)	59,632 (37.4)	
Highest	568 (18.0)	34,515 (21.7)	
Smoking status (n, %)			< 0.001^a^
Nonsmoker	2344 (74.2)	116,244 (72.9)	
Past smoker	510 (16.1)	23,320 (14.6)	
Current smoker	306 (9.7)	19,855 (12.5)	
Alcohol consumption (n, %)			< 0.001^a^
Non drinker	1804 (57.1)	81,011 (50.8)	
Past drinker	176 (5.6)	6091 (3.8)	
Current drinker	1180 (37.3)	72,317 (45.4)	
Nutritional intake			
Total calories (kcal/d)	1722.6 (574.5)	1755.6 (582.6)	0.002^a^
Protein (g/d)	57.8 (25.5)	59.7 (26.9)	< 0.001^a^
Fat (g/d)	26.5 (17.3)	28.1 (18.5)	< 0.001^a^
Carbohydrate (g/d)	309.1 (96.4)	311.8 (95.3)	0.117
Calcium (mg/d)	449.3 (287.4)	450.4 (273.1)	0.839
Phosphorus (mg/d)	878.5 (371.9)	898.6 (375.2)	0.003^a^
Potassium (mg/d)	2233.8 (1142.2)	2271.1 (1109.4)	0.069
Osteoporosis (n, %)	430 (13.6)	10,782 (6.8)	< 0.001^a^

SD, standard deviation; BMI, body mass index

^a^ Independent T-test or Chi square test. Significance at P < 0.05

In multiple logistic regression analyses, when adjusted for risk factors that showed significant association with osteoporosis in univariate analysis (i.e. age, sex, BMI, income, smoking, alcohol consumption, and nutritional intake, Additional file 1), there was a significant association of asthma with osteoporosis (Table 2). In the asthma group, there was a significant increase in osteoporosis with ORs of 1.74 (95% CI 1.55–1.94, P < 0.001) for the full-adjusted model. Sex- and age-based subgroup analyses showed comparable results.

**Table 2:** Crude and adjusted odds ratios (95% confidence interval) for osteoporosis in asthma and control groups

Characteristics	Odds ratios for osteoporosis			
	Crude	P-value	Adjusted^b^	P-value
Total participants (n = 162,579)				
Asthma	2.17 (1.96–2.41)	< 0.001^a^	1.74 (1.55–1.94)	< 0.001^a^
Control	1.00		1.00	
Age < 53 years old (n = 80,370)				
Asthma	1.81 (1.37–2.40)	< 0.001^a^	1.60 (1.20–2.12)	0.001^a^
Control	1.00		1.00	
Age ≥ 53 years old (n = 82,209)				
Asthma	1.90 (1.70–2.13)	< 0.001^a^	1.80 (1.59–2.03)	< 0.001^a^
Control	1.00		1.00	
Men (n = 55,724)				
Asthma	2.79 (1.81–4.30)	< 0.001^a^	2.17 (1.40–3.36)	0.001^a^
Control	1.00		1.00	
Women (n = 106,855)				
Asthma	2.07 (1.86–2.31)	< 0.001^a^	1.73 (1.54–1.94)	< 0.001^a^
Control	1.00		1.00	

^a^ Logistic regression model, Significance at P < 0.05

^b^ Models adjusted for age, sex, income group, BMI, smoking, alcohol consumption, and nutritional intake (total calories, protein, fat, carbohydrate, calcium, phosphorous, and potassium intake)

The adjusted OR for osteoporosis were 1.43 (95% CI 1.10–1.86, P = 0.008), 1.55 (95% CI 1.28–1.89, P < 0.001), 1.96 (95% CI 1.66–2.31, P < 0.001), in the well-controlled, being treated, and not being treated asthma groups, respectively (Table 3). We performed sex- and age-based subgroup analyses (Table 3). The adjusted ORs for osteoporosis in the subgroups of women and age ≥ 53 years were higher than those in the control group, regardless of the asthma management condition. In the subgroup of < 53 years (1.75, 95% CI 1.18–2.58, P = 0.005) and men (3.43, 95% CI 1.95–6.04, P < 0.001), the adjusted ORs for osteoporosis were higher in the not-being treated asthma group than in the control group.

**Table 3:** Crude and adjusted odds ratios (95% confidence interval) for osteoporosis according to the condition of asthma management

Characteristics	Odds ratios for osteoporosis			
	Crude	P-value	Adjusted^b^	P-value
Total participants (n = 155,506)				
Well controlled	1.71 (1.34–2.19)	< 0.001^a^	1.43 (1.10–1.86)	0.008^a^
Being treated	2.06 (1.72–2.47)	< 0.001^a^	1.55 (1.28–1.89)	< 0.001^a^
Not being treated	2.36 (2.02–2.74)	< 0.001^a^	1.96 (1.66–2.31)	< 0.001^a^
Control	1.00		1.00	
Age < 53 years old (n = 76,816)				
Well controlled	1.35 (0.72–2.54)	0.356	1.20 (0.63–2.28)	0.579
Being treated	1.88 (1.10–3.22)	0.022^a^	1.66 (0.96–2.88)	0.072
Not being treated	1.97 (1.35–2.89)	0.001^a^	1.75 (1.18–2.58)	0.005^a^
Control	1.00		1.00	
Age ≥ 53 years old (n = 78,690)				
Well controlled	1.69 (1.29–2.23)	< 0.001^a^	1.51 (1.13–2.02)	0.005^a^
Being treated	1.59 (1.31–1.93)	< 0.001^a^	1.56 (1.26–1.92)	< 0.001^a^
Not being treated	2.22 (1.87–2.63)	< 0.001^a^	2.05 (1.71–2.47)	< 0.001^a^
Control	1.00		1.00	
Men (n = 53,717)				
Well controlled	N/A	0.996	N/A	0.996
Being treated	2.30 (1.08–4.88)	0.031^a^	1.61 (0.75–3.45)	0.218
Not being treated	4.00 (2.28–7.00)	< 0.001^a^	3.43 (1.95–6.04)	< 0.001^a^
Control	1.00		1.00	
Women (n = 102,335)				
Well controlled	1.58 (1.23–2.04)	< 0.001^a^	1.49 (1.14–1.95)	0.003^a^
Being treated	2.12 (1.75–2.57)	< 0.001^a^	1.55 (1.27–1.91)	< 0.001^a^
Not being treated	2.16 (1.84–2.54)	< 0.001^a^	1.88 (1.59–2.24)	< 0.001^a^
Control	1.00		1.00	

^a^ Logistic regression model, Significance at P < 0.05

^b^ Models adjusted for age, sex, income group, BMI, smoking, alcohol consumption, and nutritional intake (total calories, protein, fat, carbohydrate, calcium, phosphorous, and potassium intake)

## Discussion

We observed a positive association of asthma with osteoporosis. Compared with the control group, the adjusted OR was highest in the not being treated group, followed by the being treated and well-controlled groups. These findings were observed in the ≥ 53 years old and women subgroups; however, the results were not significant in the < 53 years old and men subgroups of the being treated and well-controlled groups.

Several asthma-related factors are associated with osteoporosis development. First, inflammatory cytokines, including TNF-α and IL-6, are associated with an increased risk of asthma [18, 19]. TNF-α and IL-6 have been shown to mediate inflammation, which contributes to bone resorption and osteoporosis through stimulation of osteoclast development and activity [20, 21]. Second, increased inflammatory cytokine release can result in anorexia [22]. Therefore, patients with chronic inflammation are exposed to malnutrition, which leads to osteoporosis [23]. Third, there is a high prevalence of vitamin D deficiency among patients with asthma. A systematic review and meta-analysis reported that the prevalence of vitamin D deficiency was significantly greater among patients with asthma than among controls (relative risk = 1.59, 95% CI 1.07–2.36) [24]. Vitamin D deficiency causes secondary hyperparathyroidism; further, parathyroid hormone causes osteoblast activation. This stimulates preosteoclast transformation into mature osteoclasts, which causes osteopenia and osteoporosis, as well as increase the fracture risk [25]. Fourth, adults with asthma are less physically active compared with healthy individuals [26, 27] given that exercise and physical activities can trigger bronchoconstriction, which causes exertional dyspnea and other respiratory symptoms. Physical activity is an important factor for bone loss and osteoporosis prevention throughout an individual’s life [28]. Finally, there have been several reports of corticosteroid-induced osteoporosis. Corticosteroids (oral and inhaled) are the mainstay asthma management regimens due to their anti-inflammatory effect. They have been shown to reduce osteoblast function and proliferation, increase osteoblast and osteocyte apoptosis, and prolong the lifespan of osteoclasts [29]. Therefore, the correlation between asthma and osteoporosis could be secondary to the steroid treatment in patients with asthma.

We found that the ORs for osteoporosis were highest (1.96) in the not being treated asthma group. The correlation between asthma and osteoporosis could be dependent on the status of asthma management. Poor asthma management is associated with several factors, which may be associated with osteoporosis development. First, uncontrolled asthma is associated with an increased systemic inflammatory state. A study on cytokines in bronchoalveolar lavage fluid reported that patients with active asthma had increased TNF-α and IL-6 levels than healthy controls and stabilized patients with asthma [30]. Second, low circulating levels of 25-hydroxyvitamin D, which is the major circulating vitamin D metabolite, was associated with poor asthma management [31]. A meta-analysis reported that vitamin D supplementation reduced the rate of asthma exacerbation requiring treatment with systemic corticosteroids (adjusted incidence rate ratio = 0.74, 95% CI 0.56–0.97, P = 0.03) [32]. Third, low physical activity was found to be associated with poor asthma management. In Netherlands [27], a study divided patients with asthma according to their Asthma Control Questionnaire category and reported significant between-group differences in the physical activity level [1.58 ± 0.18 (well controlled) vs. 1.54 ± 0.13 (partially controlled) vs. 1.49 ± 0.13 (uncontrolled), P = 0.002], steps/day [8169 ± 3225 (well controlled) vs. 8244 ± 2621 (partially controlled) vs. 6712 ± 3153 (uncontrolled), P = 0.005]; and time spent at vigorous exercise (> 6 metabolic equivalents) [27 ± 22 (well controlled) vs. 22 ± 13 (partially controlled) vs. 14 ± 13 min/day (uncontrolled), P < 0.001].

The ORs for osteoporosis were higher in the being treated asthma group (1.55) than in the well-controlled asthma group (1.43). This could be attributed to glucocorticoid-induced osteoporosis; however, the effect of inhaled corticosteroids (ICSs) on bone mineral density (BMD) in patients with asthma remains unclear [33, 34]. Moreover, oral corticosteroid treatment is known to reduce BMD and increase fracture risk [35]. On the other hand, some studies have reported an independent asthma effect on osteoporosis regardless of steroid treatment [14, 36]. A Korean healthcare center-based study reported a significant BMD reduction (–0.53 ± 1.50 in the lumbar spine; − 0.46 ± 0.97 in the femur) in patients with positive AHR test results without a history of systemic corticosteroid use [14]. In a USA hospital-based study, patients with chronic lung disease (including chronic obstructive pulmonary disease [COPD] and asthma) who never had glucocorticoid treatment had an almost fourfold prevalence of osteoporosis compared with the control group [36]. Moreover, the well-controlled asthma group showed a significant correlation of asthma with osteoporosis. This could be attributed to the fact that asthma or the effects of previously used corticosteroids could be potential risk factors for osteoporosis.

In the women and age ≥ 53 years-old subgroups, there was a correlation found between asthma and osteoporosis regardless of the asthma management condition. Aging is also associated with an increased risk of osteoporosis [37]. Moreover, women are at increased estrogen-related osteoporosis risk since estrogen is crucially involved in bone growth and maturation, as well as in bone turnover regulation in adults [38]. Contrastingly, in the men and < 53 years-old subgroups, there was a significant positive association between asthma and osteoporosis in the not being treated asthma group only. Although we did not obtain information regarding menopause and hormonal replacement treatment in women, the rate of pre-menopausal women is likely to be higher at < 53 years old than in the older age subgroup. Moreover, the effect of non-treated asthma on osteoporosis was higher in men (OR = 3.43) than in women (OR = 1.88), which could be attributed to other factors, including systemic inflammation and physical activity, being more in apparent men than in women.

This study has several limitations. First, the disease history was based on self-reported questionnaires, which may have a possibility of recall bias or under/over-reporting of the disease. However, self-reported disease history using questionnaires is widely accepted in large-scale population-based epidemiologic studies and structured questions were asked by trained interviewers in this cohort. In addition, a recent Korean study using the National Health Insurance Sharing Service database 2002–2015 showed that the prevalence of asthma ranged from 1.55% to 2.21% [39], which was similar to the prevalence of asthma in our results (1.94%). Second, the asthma management condition was also determined using a questionnaire. We could not assess the actual medication use, including oral corticosteroid and ICS. However, this was a large-scale population-based study; moreover, patients with asthma were divided to the well-controlled and not being treated asthma groups. Although none of the groups was treated with steroids, our findings indicate that untreated and neglected patients with asthma are at higher risk of osteoporosis. Third, we did not obtain information regarding asthma onset and duration, other comorbidities such as kidney disease, malabsorption, inflammatory bowel disease, and autoimmunity, serum vitamin D levels, other medications, and hormonal replacement treatment. However, we adjusted for nutritional intake, including total calories, protein, fat, carbohydrate, calcium, phosphorous, and potassium intake, since nutrition is an important modifiable factor that affects bone health [40]. Lastly, inflammatory cytokine levels were not measured. There is a need for further studies to determine the effects of systemic inflammatory response on osteoporosis development in patients with asthma.

## Conclusion

Asthma was associated with osteoporosis in the Korean adult population. Patients with untreated asthma showed the highest ORs for osteoporosis. Clinicians should be aware of osteoporosis in patients with asthma, especially in those who are untreated and neglected.

## Supplementary information

**Additional file 1: Table S1.** General characteristics of participants according to the osteoporosis.

## Data Availability

Data in this study were from the Korean Genome and Epidemiology Study (KoGES; 4851-302), National Research Institute of Health, Centers for Disease Control and Prevention, Ministry for Health and Welfare, Republic of Korea.

## Abbreviations

TNF: Tumor necrosis factor
IL: Interleukin
AHR: Airway hyper-responsiveness
OR: Odds ratio
CI: Confidence interval
KoGES: Korean Genome and Epidemiology Study
HEXA: Health examinee
BMI: Body mass index
ICS: Inhaled corticosteroid
BMD: Bone mineral density
COPD: Chronic obstructive pulmonary disease
