# Supplementary material for: The association of asthma and its subgroups with osteoporosis: a cross-sectional study using KoGES HEXA data
Source: Allergy Asthma Clin Immunol. 2020 Sep 25;16:84. doi: 10.1186/s13223-020-00482-6 (PMC7519551; doi:10.1186/s13223-020-00482-6)
Supplement: Supplementary file 1 — Additional file 1: Table S1. General characteristics of participants according to the osteoporosis. [file 13223_2020_482_MOESM1_ESM.docx]

**Table S1** General characteristics of participants

| Characteristics | | Total participants | | P-value |
| --- | --- | --- | --- | --- |
|  |  | Osteoporosis | Non-osteoporosis |  |
| Age (mean, SD, y) | | 59.1 (7.0) | 52.8 (8.3) | <0.001^a^ |
| Sex (n, %) | |  |  | <0.001^a^ |
|  | Men | 483 (4.3) | 55,241 (36.5) |  |
|  | Women | 10,729 (95.7) | 96,126 (63.5) |  |
| BMI (mean, SD, kg/m^2^) | | 23.6 (2.9) | 24.0 (2.9) | <0.001^a^ |
| Income (n, %) | |  |  | <0.001^a^ |
|  | Missing, no response | 1,991 (17.8) | 18,802 (12.4) |  |
|  | Lowest | 4,703 (41.9) | 41,372 (27.3) |  |
|  | Middle | 3,156 (28.1) | 57,472 (38.0) |  |
|  | Highest | 1,362 (12.1) | 33,721 (22.3) |  |
| Smoking status (n, %) | |  |  | <0.001^a^ |
|  | Nonsmoker | 10,546 (94.1) | 108,042 (71.4) |  |
|  | Past smoker | 370 (3.3) | 23,460 (15.5) |  |
|  | Current smoker | 296 (2.6) | 19,865 (13.1) |  |
| Alcohol consumption (n, %) | |  |  | <0.001^a^ |
|  | Non drinker | 8,356 (74.5) | 74,459 (49.2) |  |
|  | Past drinker | 282 (2.5) | 5,985 (4.0) |  |
|  | Current drinker | 2,574 (23.0) | 70,923 (46.9) |  |
| Nutritional intake | |  |  |  |
|  | Total calories (kcal/d) | 1645.3 (566.4) | 1763.0 (582.9) | <0.001^a^ |
|  | Protein (g/d) | 55.0 (26.2) | 60.0 (26.9) | <0.001^a^ |
|  | Fat (g/d) | 23.7 (17.0) | 28.4 (18.5) | <0.001^a^ |
|  | Carbohydrate (g/d) | 299.0 (94.2) | 312.7 (95.3) | <0.001^a^ |
|  | Calcium (mg/d) | 453.4 (285.2) | 450.1 (272.5) | 0.224 |
|  | Phosphorus (mg/d) | 853.5 (378.8) | 901.6 (374.8) | <0.001^a^ |
|  | Potassium (mg/d) | 2,168.3 (1,135.1) | 2,277.9 (1,107.9) | <0.001^a^ |
| Asthma (n, %) | | 430 (3.8) | 2,730 (1.8) | <0.001^a^ |
| Asthma treatment (n, %) | |  |  | <0.001^a^ |
|  | Non-asthma | 10,597 (96.3) | 141,910 (98.2) |  |
|  | Well controlled | 72 (0.7) | 564 (0.4) |  |
|  | Being treated | 137 (1.2) | 889 (0.6) |  |
|  | Not being treated | 200 (1.8) | 1,137 (0.8) |  |

SD: standard deviation, BMI: body mass index

* Independent T-test or Chi-square test. Significance at P < 0.05
